# Comparative genomics reveals *Lysinibacillus sphaericus* group comprises a novel species

**DOI:** 10.1186/s12864-016-3056-9

**Published:** 2016-09-05

**Authors:** Camilo Gómez-Garzón, Alejandra Hernández-Santana, Jenny Dussán

**Affiliations:** Centro de Investigaciones Microbiológicas (CIMIC), Universidad de los Andes, Cra 1 N. 18 A-12, Bogotá, Colombia

**Keywords:** *Lysinibacillus sphaericus*, Pan-genome, Core-genome, Phylogeny, Larvicidal

## Abstract

**Background:**

Early in the 1990s, it was recognized that *Lysinibacillus sphaericus*, one of the most popular and effective entomopathogenic bacteria, was a highly heterogeneous group. Many authors have even proposed it comprises more than one species, but the lack of phenotypic traits that guarantee an accurate differentiation has not allowed this issue to be clarified. Now that genomic technologies are rapidly advancing, it is possible to address the problem from a whole genome perspective, getting insights into the phylogeny, evolutive history and biology itself.

**Results:**

The genome of the Colombian strain *L. sphaericus* OT4b.49 was sequenced, assembled and annotated, obtaining 3 chromosomal contigs and no evidence of plasmids. Using these sequences and the 13 other *L. sphaericus* genomes available on the NCBI database, we carried out comparative genomic analyses that included whole genome alignments, searching for mobile elements, phylogenomic metrics (TETRA, ANI and *in-silico* DDH) and pan-genome assessments. The results support the hypothesis about this species as a very heterogeneous group. The entomopathogenic lineage is actually a single and independent species with 3728 core genes and 2153 accessory genes, whereas each non-toxic strain seems to be a separate species, though without a clear circumscription. Toxin-encoding genes, *binA, B* and *mtx1, 2, 3* could be acquired via horizontal gene transfer in a single evolutionary event. The non-toxic strain OT4b.31 is the most related with the type strain KCTC 3346.

**Conclusions:**

The current *L. sphaericus* is actually a *sensu lato* due to a sub-estimation of diversity accrued using traditional non-genomics based classification strategies. The toxic lineage is the most studied with regards to its larvicidal activity, which is a greatly conserved trait among these strains and thus, their differentiating feature. Further studies are needed in order to establish a univocal classification of the non-toxic strains that, according to our results, seem to be a paraphyletic group.

**Electronic supplementary material:**

The online version of this article (doi:10.1186/s12864-016-3056-9) contains supplementary material, which is available to authorized users.

## Background

Since the discovery of entomopathogenic activity in *Bacillus thuringiensis* in the 1960s, many bacteria with insecticidal activity have been described. Isolates of *B. thuringiensis* and *Lysinibacillus sphaericus* are frequently reported [1]. The latter is more active against *Culex* and *Anopheles* spp. and more persistent in polluted aquatic environments than *B. thuringiensis* var. *israelensis* [2, 3]. *Lysinibacillus sphaericus* is a gram-positive and spore-forming bacteria isolated for the first time from fourth-instar larvae of *Culiseta incidens* near Fresno, California [4]. However, this strain displayed a low level of toxicity [5] and it was not until the 1970s that the first strains with potential use as mosquito-control agents were discovered [6].

In spite of being widely used in biological control programs, not all strains of *L. sphaericus* are toxic against mosquitoes. Nowadays, it is well known that a plethora of insecticidal toxins are responsible for the entomopathogenic activity of the toxic strains. Binary prototoxin (Bin) is the major insecticidal protein produced by *L. sphaericus;* it is contained inside the parasporal crystal and comprises two proteins: BinA (42 kDa) and BinB (51 kDa). After being ingested by larva, these proteins are solubilized in the gut and undergo proteolysis to active lower molecular weight derivatives [2, 7, 8]. Other crystal proteins, Cry48 and Cry49, might be produced on sporulation by some toxic strains. These toxins are related to Cry toxins of *B. thuringiensis* and Bin family toxins, respectively [1]. *L. sphaericus* may also produce insecticidal toxins during vegetative stage; this is the case of Mtx proteins [9, 10] whose mode of action remains to be elucidated.

Formerly known as *Bacillus sphaericus*, *L. sphaericus* is characterized by having a spherical terminal spore and by its inability to utilize carbohydrates, except N-acetylglucosamine [11]. Instead, it uses organic and amino acids as carbon sources [5]. This species may be found in soil and aquatic environments and, recently, has gained attention because it has shown outstanding potential for environmental and industrial applications beyond biological control, especially in bioremediation of toxic metals [12–14], phosphorous solubilization [15], among others [16].

In 2007, this species was reclassified to a new genus according to phenotypic traits, mainly based on differences in peptidoglycan composition which includes lysine and aspartic acid instead of *meso*-diaminopimelic acid, the major component of *Bacillus* cell wall [17]. No genomic support to assess this classification was reported until a few years ago, when Hu and coworkers investigated the phylogenetic relationship between four toxic and three non-toxic strains. Their findings suggested a new species for insecticidal strains and provided evidence for toxicity evolution by means of horizontal gene transfer (HGT) [18]. However, a more comprehensive analysis is required as the number of available genome sequences has doubled. Therefore, we aimed to perform a broader evaluation of the intraspecific genetic diversity of *L. sphaericus* as species and as mosquito-control agent.

## Results

### A new *L. sphaericus* genome is now available

The genome of *L. sphaericus* OT4b.49, a previously isolated Colombian strain [14], was sequenced using Pacific Biosciences technology and assembled, obtaining 3 contigs (4.6 Mbp, 29.6 Kbp and 14.9 Kbp) and no evidence of plasmids. The estimated coverage was 242 × with 981,395,594 bp produced, of which 11,921,224 bp were from circular consensus sequencing (CCS). Moreover, the GC content (37.30 %), predicted genes (4486) and genome size (4.7 Mbp) were expected according to the currently reported genomes. This newly available genome together with the other 13 *L. sphaericus* genomes previously uploaded to the NCBI database, were used in the current study (Table 1).

**Table 1:** *L. sphaericus* genomes used in this study

Strain	Toxicity^a^	Level	Genome size (bp)	Contigs	Accession no.	Reference
C3-41	High	Complete	4,639,821	2^b^	CP000817	[48]
2362	High	Complete	4,692,801	1	CP015224	[49]
III(3)7	High	Complete	4,663,526	2^b^	CP014856	[14]
OT4b.25	High	Complete	4.665,575	2^b^	CP014643	[50]
OT4b.49	High	Draft	4,668,840	3	LWHI01000000	This study
CBAM5	High	Draft	5,156,460	93	AYKQ00000000	[13]
LP1-G	High	Draft	4,542,839	143^b^	JPDL01000000	[17, 51]
2297	Medium	Draft	4,516,760	278	JPDJ01000000	[18]
SSII-1	Low	Draft	4,651,985	138	JPDK01000000	[18]
1987	Non-toxic	Draft	4,906,630	70	JMMU01000000	Not published
OT4b.31	Non-toxic	Draft	4,856,302	94	AQPX00000000	[52]
B1-CDA	Non-toxic	Draft	4,509,276	84	LJYY01000000	[12]
KCTC 3346	Non-toxic	Draft	4,560,870	83	AUOZ00000000	[53]
NRS 1693	Non-toxic	Draft	4,603,690	546	JPDM01000000	[18]

^a^High: presence of *binA*, *B* and *mtx*1, 2, 3 or *cry48*, *49*; Medium: Only *binA, B* and *mtx2*; Low: only *mtx2.* This classification was previously established by Ge et al. by associating the presence of toxin genes with mosquitocidal activities of 35 *L. sphaericus* isolates [54]

^b^At least one plasmid-associated contig

### 16S rDNA homology cannot differentiate toxic from non-toxic isolates

**Fig. 1:**
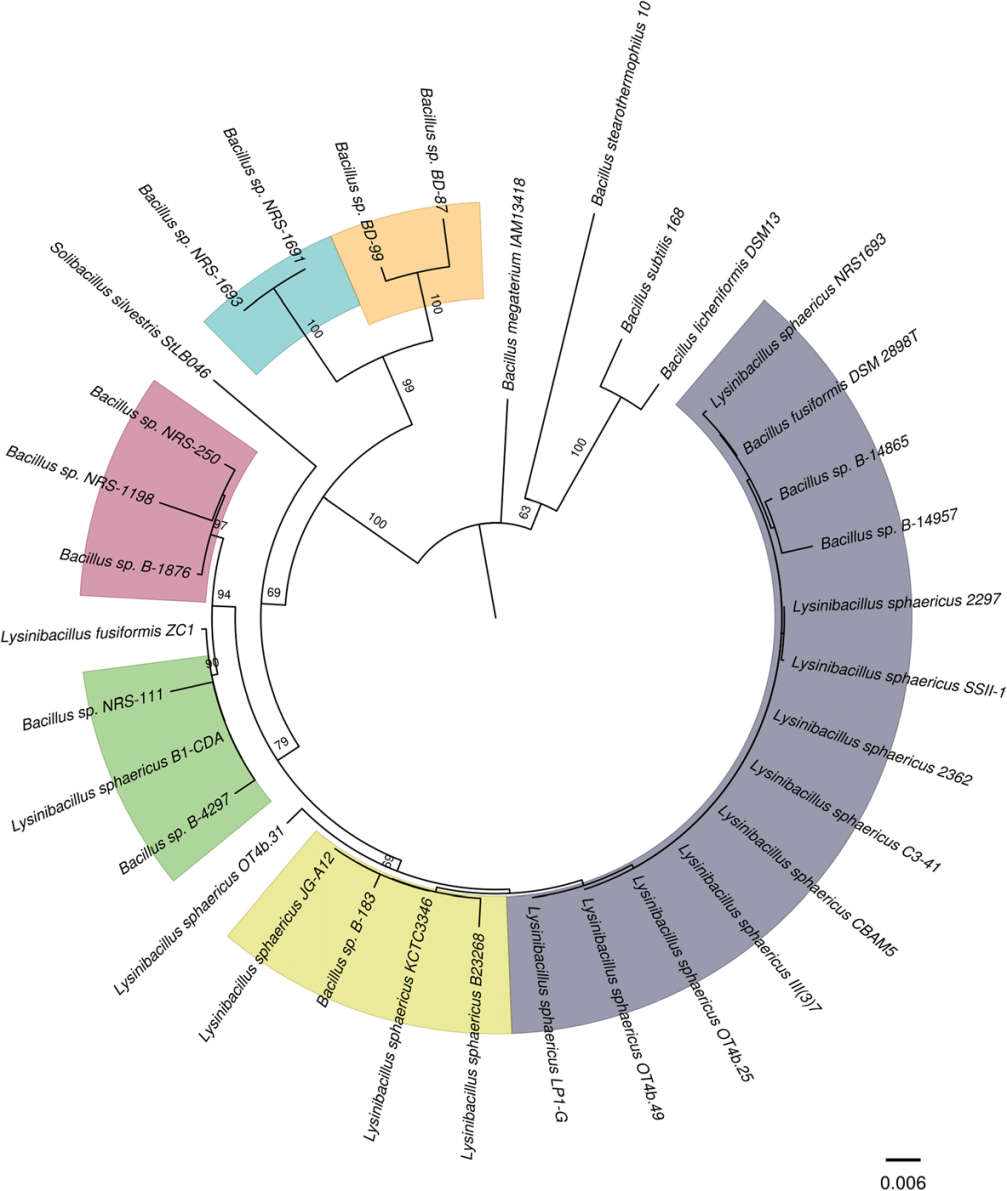
Phylogenetic tree of round-spored bacilli showing the current 16S rDNA-based taxonomy. *L. sphaericus* strains can be found in three out of the six highlighted homology groups. All the toxic strains are clustered in the group in purple, however, not all the strains in that group are toxic. Bootstrap values for 500 replicates are shown in the branches

*L. sphaericus* is generally classified into five DNA-homology groups based on the similarity of 16S rDNA sequences. These groups are also related by some phenotypic traits and molecular markers [19]. We reconstructed the 16S rDNA phylogeny including the 14 *L. sphaericus* strains herein analyzed and found the same clustering pattern reported in previous studies [19, 20] with high bootstrap support. All the toxic strains are grouped together in the same lineage with some non-toxic strains and, separately, the other non-toxic strains are distributed among the other groups (Fig. 1). Therefore, as it was suggested, only in the view of 16S rDNA homology, toxicity does not seem to be appropriate as the sole differentiator [21]. However, further analyses revealed high diversity within *L. sphaericus* and suggested that toxicity is a differentiating feature that could be acquired in a single evolutionary event.

### Toxic strains comprise a nearly clonal and independent lineage with a high degree of synteny

**Fig. 2:**
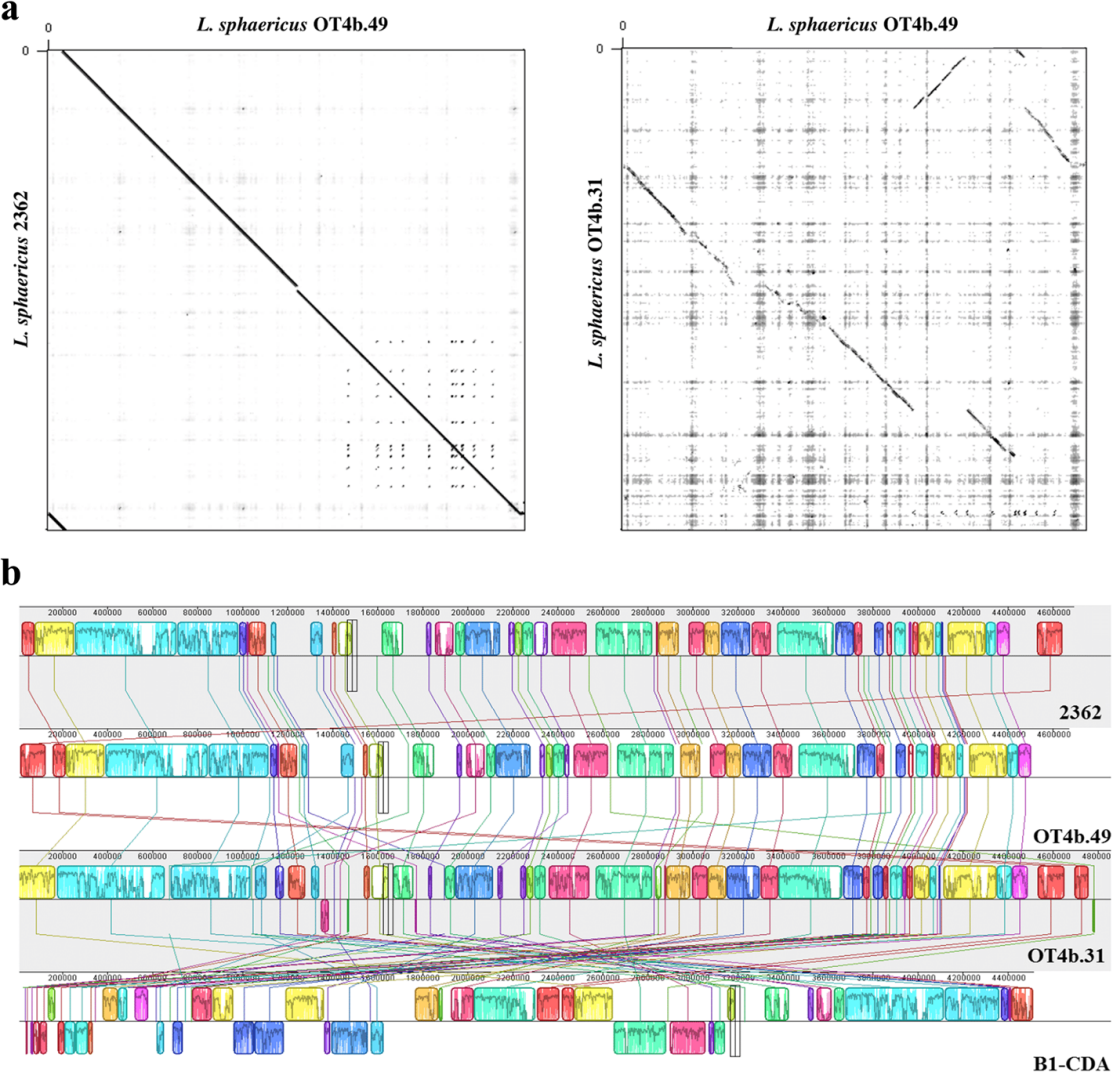
Whole genome alignments between toxic and non-toxic strains. **a** Dot-plots of nucleotide identities of the toxic strains OT4b.49 against 2362 (*left*) and OT4b.49 against the non-toxic strain OT4b.31 (*right*). **b** Nucleotide-based alignment of the genomes from two toxic (OT4b.49 and 2362) and two non-toxic (OT4b.31 and B1-CDA) strains. Homologous blocks are shown as identically colored regions and linked across the genomes. Regions that are inverted relative to *L. sphaericus* 2362 are shown below the central axis of each sequence

We performed comparative genomic analyses which supported the hypothesis of the toxic strains as an independent group within *L. sphaericus*. The first evidence came from multiple genome alignments and the evaluation of genomic architectures carried out by Gepard [22] and MAUVE [23] softwares. The toxic strains exhibited strong syntenic relationships, though some rearrangements, mainly duplications, were detected (Fig. 2a). On the other hand, genomes from non-toxic strains showed several differences among them and in comparison to the toxic strains genomes, especially the strain B1-CDA, in which several inversions were identified (Fig. 2b).

**Fig. 3:**
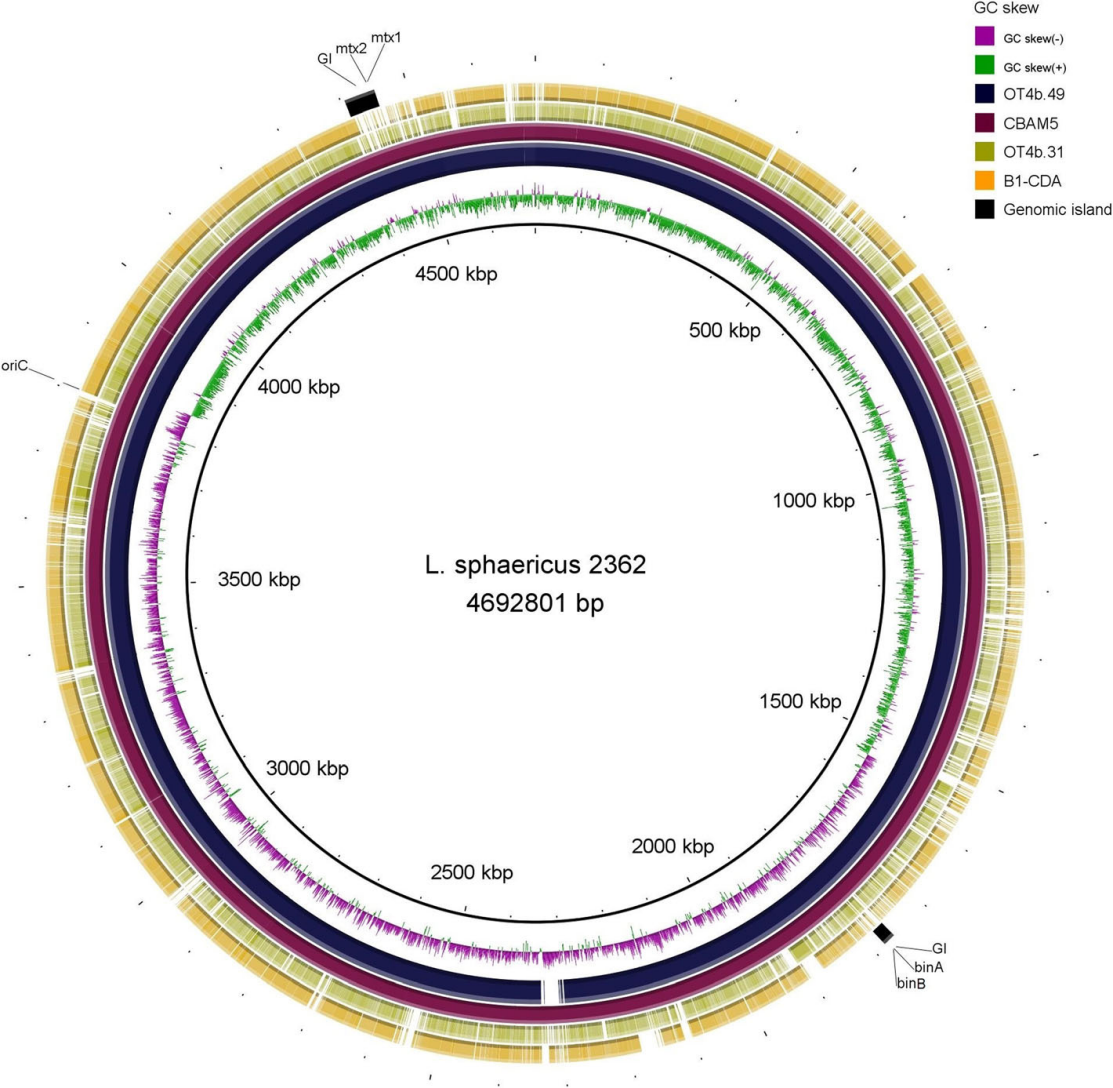
Circular map that compares genomes of *L. sphaericus* OT4b.49, CBAM5, OT4b.31, and B1-CDA against 2362. Each circle represents the genome from one strain, and the colored blocks in it represent sequences with >90 % identity relative to *L. sphaericus* 2362. The GIs in the immediacy of encoding toxin genes as well as the origin of replication are spotted. GC skew is shown in the inner circle

An additional whole genome alignment, based on BLAST, was conducted and visualized using BRIG [24]. The ring image obtained clearly showed the high similarity between the toxic strains and a great heterogeneity when they are compared to the non-toxic ones (Fig. 3). Intriguingly, when this assay was performed with a toxic strain as a reference (as shown in Fig. 3), the divergence pattern among the non-toxic strains was very similar. This suggests the existence of gene clusters unique for the toxic strains beyond the toxin-encoding genes.

### HGT might have played a role in toxicity acquisition

Eleven Genomic Islands (GIs) were detected for the strain OT4b.49. As reported by Hu and coworkers, the identified GIs comprise sequences of mobile genetic elements as prophages and transposons, and several recombination-involved proteins as integrase, recombinase, and transposase [18]. Interestingly, mosquitocidal toxin coding genes are within or in the immediacy of those GIs (Fig. 3). All of the completed genomes from toxic strains were evaluated for GIs and, as it was previously hypothesized, all of them have between 7 and 11 GIs associated with the toxin genes. This suggests a role for these mobile large segments of DNA in the acquisition of entomopathogenic activity.

### The toxic lineage appears to represent a novel species

**Fig. 4:**
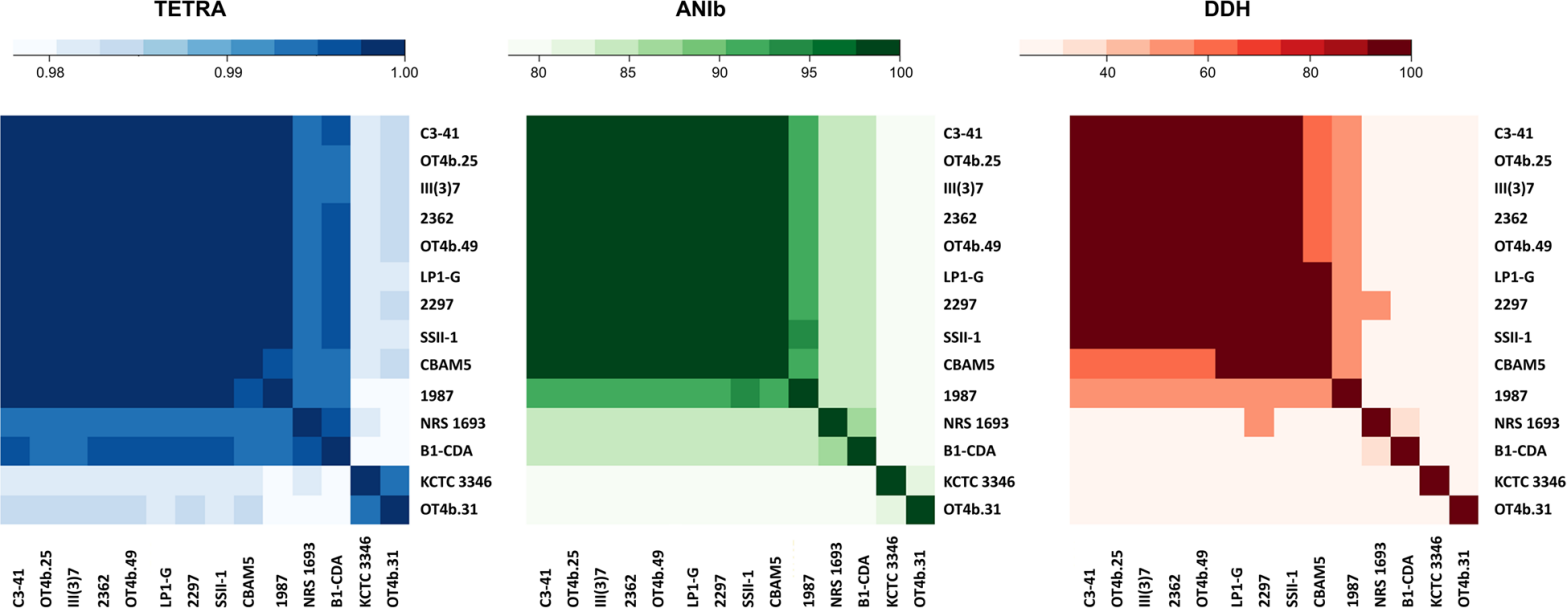
Heatmaps representing metrics for the evaluation of species circumscription among *L. sphaericus* strains. The extent of nucleotide identity was calculated according to different indices for species circumscription: TETRA, ANIb and DDH as illustrated. The key color is shown for each figure

Since some researchers have proposed that *L. sphaericus* could compromise of more than one species in a single *sensu lato* [18–20], we calculated the correlation indexes of tetranucleotide signatures (TETRA) and the Average Nucleotide Identity based on BLAST and MUMmer (ANIb and ANIm) as metrics to assess the species circumscription [25]. In the same way, we performed in-silico genome-to-genome comparison to calculate digital DNA:DNA hybridization estimates (DDH) [26]. All the evaluated metrics allowed a clearer circumscription for the toxic lineage, providing support to the hypothesis of this group as a novel species. On the contrary, it is not clear if all the non-toxic strains belong to the same species or not because, although some identity values were below the threshold of species level, the results were not consistent across the metrics (Fig. 4). For instance, TETRA values indicated that the strains 1987, B1-CDA and NRS 1693 are right on the species boundary with the toxic group, in contrast to results from ANIb which suggested they are different species from each other. Besides the consensus for the toxic group, the three metrics designated the strains OT4b.31 and KCTC 3346 (*L. sphaericus* type strain) as the most divergent ones.

Furthermore, to gain a deeper insight on this matter, we aimed to reveal distinctive traits of toxic and non-toxic strain by identifying, evaluating, and comparing clusters of orthologous genes (COGs) from protein sequences comparisons*.* In concordance with results mentioned above, little functional diversity was found in 4 representatives of toxic lineage since no unique genes were detected for any strain (Additional file 1: Figure S1). However, when the same evaluation was carried out with two toxic and two non-toxic strains, a greater heterogeneity was observed, with the toxic strains being the ones which shared the highest amount of COGs (Additional file 1: Figure S1). The core and accessory genes of *L. sphaericus* as well as core and accessory genes of toxic lineage were identified by following Roary pipeline [27]. Three thousand seven hundred and twenty eight core genes (defined as genes in more than 95 % of evaluated strains) were found in toxic lineage as well as a pan-genome pool containing 5881 genes. In sharp contrast, only 391 genes constituted the core-genome and 20,217 genes constituted the pan-genome when both toxic and non-toxic strains were evaluated.

**Fig. 5:**
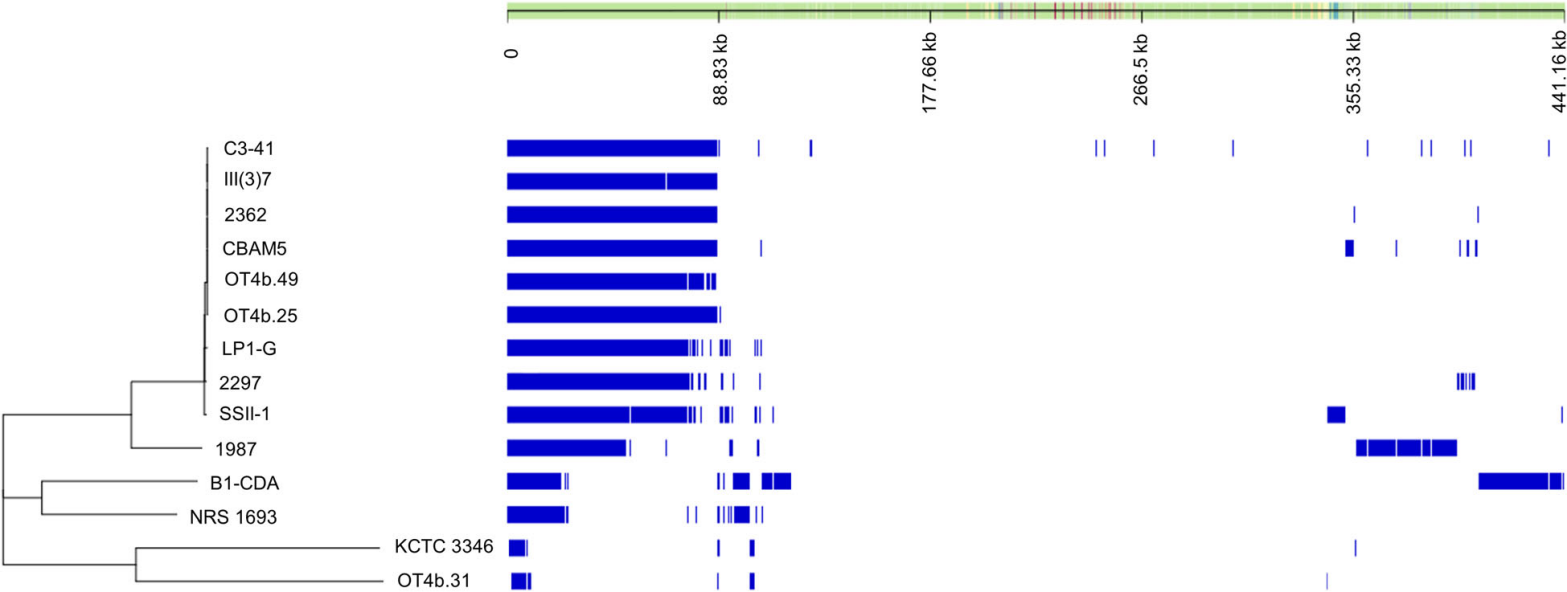
Core and accessory genes of *L. sphaericus* genomes. The upper panel shows both contigs and annotated genes which are inferred from pan-genome content and might not represent the genome order. Genes are represented and mapped as blue blocks. Genes shared by two or more sequences are mapped in the same position. The phylogenetic tree on the left panel was constructed by FastTree 2.0 based on the core genes alignment obtained from Roary

The phylogenetic tree constructed based on core genes supports the hypothesis about toxic strains as separate species from non-toxic strains, the latter without a clear circumscription (Fig. 5). Interestingly, the strain OT4b.31 was the most related with the strain KCTC 3346, which is the type strain for *L. sphaericus* [28]. Thus, this group would be *L. sphaericus sensu stricto* whereas toxic lineage should be considered as a new species.

**Fig. 6:**
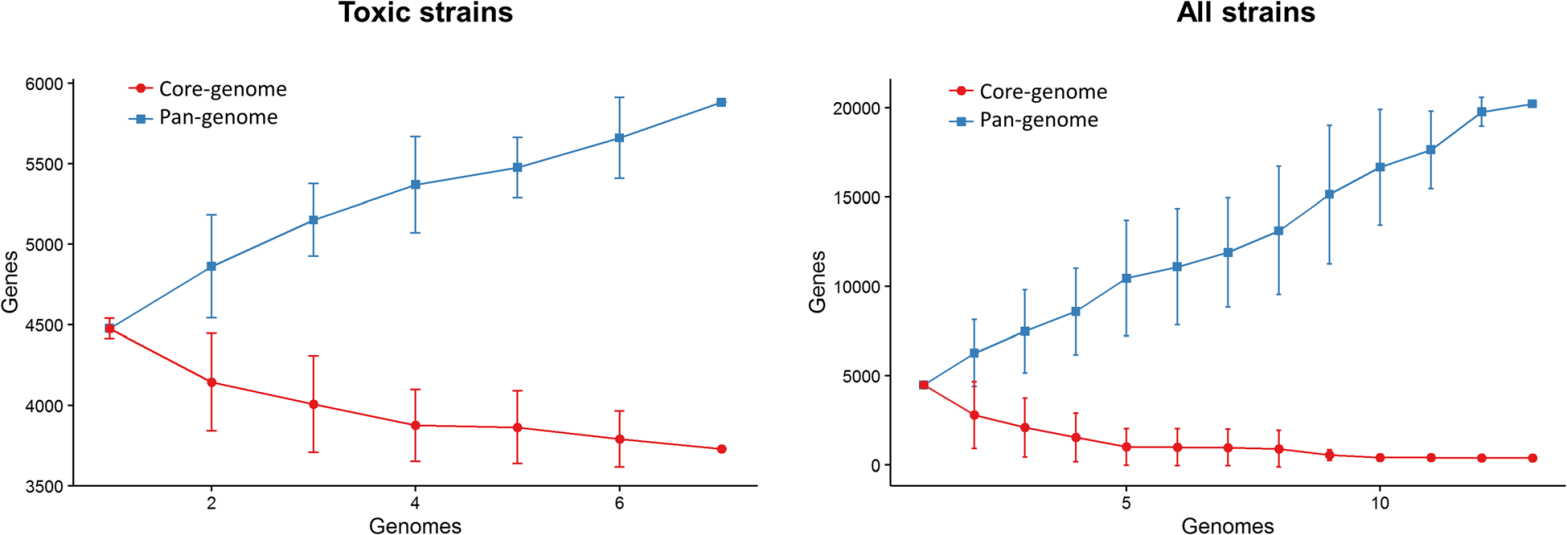
Pan- and core-genome of *L. sphaericus*. The curves depict the pan and core-genome, for toxic strains and for the complete set of analyzed strains, both as function of the number of genomes

Finally, we assessed the pan-genome of both the toxic lineage and *L. sphaericus* as it is described nowadays (comprising toxic and non-toxic strains). The pan-genome of the toxic lineage of *L. sphaericus* was composed of 3728 core genes and 2153 accessory genes, whereas the pan-genome of the whole *L. sphaericus species* was larger, with 391 core genes and 19,826 accessory genes, which provides evidence for high intra-species diversity (Fig. 6 and Additional file 2: Figure S2).

## Discussion

It is commonly recognized that a few sequenced genomes may misrepresent the entire genetic repertoire of a species [29, 30]. That is why the current availability of 14 *L. sphaericus* genomes has made this traditionally controversial group an excellent candidate for phylogenomics and pan-genomic studies that clarify the species boundaries for this taxon. In this work, we carried out a comprehensive analysis of *L. sphaericus* as a species and mosquito-control agent, obtaining results that suggest the need of a new species designation.

The results showed a high diversity within *L. sphaericus*, with entomopathogenicity being the main feature that allows a clear distinction among the strains. This supports previous studies whereby a reevaluation of *L. sphaericus* as a species was suggested. By convention, round-spored mesophilic bacilli that grow at neutral pH and are unable to ferment carbohydrates have been classified as *L. sphaericus sensu lato* [20]. As unique phenotypic traits are discovered, novel species have been designated from this group, this is the case for *L. fusiformis, L. boronitolerans,* and *Sporosarcina globispora,* among others [19]. As Nakamura states, the dependence of early studies on insensitive methods hindered estimation of diversity and fostered the creation of heterogeneous species that includes toxic and non-toxic strains [19]. Hence, variability in toxicity might arise from genetic variability and incorrect classification.

We found evidence that suggests the toxicity could have been acquired by a HGT event because toxin genes were found flanked by genomic islands containing several integrase, recombinase, and transposase sequences. However, we are still not able to clarify from what kind of gene transfer event the mosquitocidal activity arose.

It is very intriguing that all toxic strains shape a nearly clonal group in spite of their very different provenance: strains 2362, C3-41 and OT4b.49 were respectively isolated from Africa, Asia and South America. Therefore, we hypothesize that toxins acquisition lead to the emergence of the toxic lineage by providing a fitness increase and thus, a great genomic stabilization.

Since the toxic lineage are made up by nearly clonal strains, the sole presence of *binA* or *binB* (which are always together) and *mtx2* (or *cry*) toxin-encoding genes is a good indicator of the feasibility for using a round-spored bacilli as mosquito-control agent. This could be easily assessed, for instance, by a PCR assay. In addition, these genes also would indicate the presence of other interesting core genes from this lineage, such as those that encode for S-layer protein and metal efflux pumps [14, 31].

Herein, we compared 14 *L. sphaericus* genomes with one another by using ANIb, TETRA, and digital DDH, in order to achieve a clearer species circumscription [25, 26, 32]. The results certainly showed the toxic lineage of *L. sphaericus* as a single and independent species (Fig. 4 and Additional file 3: Table S1). A further evaluation of remaining members of the *L. sphaericus* species is required due to values outside the intra-species range (<96 % and <0.999 for ANI and TETRA, respectively, and <70 % for DDH) in some non-toxic strains.

An alternative and novel way to describe a bacterial species is by its pan-genome, which is the sum of the core (genes present in all strains), dispensable (genes present in two or more strains), and unique (genes present in single strains) genomes [30]. As Tettelin and coworkers proposed, by defining the pan-genome of a bacterium, insights both on its biology and life style can be gained as well as implications for the definitions of the species itself [33]. Our comparative analysis of 14 *L. sphaericus* genomes indicated a pan-genome with frequent rearrangements, revealing the striking genomic heterogeneity inside this group. When performing the same comparative analysis on the 8 entomopathogenic strains, an open but smaller pan-genome as well as highly syntenic regions and less frequent genomic rearrangements were found (Fig. 6).

Finally, it is important to take into account that frequent gaps and sequencing errors might cause underestimation in genome annotation and therefore, errors in the estimation of the core- and pan-genome [34]. Only 4 of the 14 genomes analyzed are completed as a single chromosomal contig, which constitutes an inherent limitation of this study and highlights the importance of technologies that make closed genomes possible.

## Conclusions

We generated a draft genome for the Colombian mosquitocidal *L. sphaericus* OT4b.49 and carried out an analysis of the full repertoire of *L. sphaericus* available genomes in order to assess intraspecific diversity.

The current study provides strong evidence for considering the toxic-lineage of *L. sphaericus* as a new species. Historically, many round-spored mesophilic bacilli have been grouped under *L. sphaericus* classification, leading to the formation of a heterogeneous *sensu lato*. We assessed taxonomic composition by means of overall genome relatedness indices and phylogenomic analysis based on core genes. We found that toxic strains form a well-defined lineage that should be considered as a novel species. The differentiating feature of this species is the presence of toxin-encoding genes such as *binA, B* and *mtx1, 2, 3*, which might be acquired by HGT.

On the other hand, the remaining *L. sphaericus* strains did not show a clear circumscription and are, indeed, a paraphyletic group. Further studies are needed in order to establish a univocal classification, though this is still challenging in the light of the absence of an unambiguous species definition for bacteria.

## Methods

### Genome sequencing and assembly

The genome sequencing of *L. sphaericus* OT4b.49 was carried out using Pacific Biosciences technology with 1 SMRT cell, P4-C2 chemistry, and a mixed library (CCS and subreads). This service was provided by McGill University and Génome Québec Innovation Centre. Contig assembly was done using the HGAP 2.0 workflow [35]. Sequencing errors were corrected by aligning multiple short reads on longer reads. Subsequently, the corrected reads were used as seeds into Celera Assembler [36] to obtain contigs. These contigs were polished through an alignment of raw reads on contigs by BLASR [37] and then, high quality consensus sequences were generated from these contigs by a variant calling algorithm (Quiver).

### Genome annotation

The genome of *L. sphaericus* OT4b.49 was annotated using the NCBI Prokaryotic Genome Annotation Pipeline [38] and RAST [39]. In addition, the 14 genomes used in this study (Table 1) were re-annotated by Prokka [40], which locates ORFs by Prodigal and RNA regions using RNAmmer, Aragorn, SignalP and Infernal. Then, it annotates the translated sequences by a homology searching with BLAST and HMMER, followed by a searching against public databases (CDD, PFAM, and TIGRFAM) and the Prokka “Kingdom Bacteria” database.

### 16S rDNA phylogeny

The 16S rDNA sequences obtained with Prokka, together with the 16S rDNA sequences from other bacilli listed below, were aligned using MEGA 6.0 [41] with the MUSCLE algorithm. The phylogenetic tree was then constructed by the neighbor-joining method and the distances, computed with the Kimura’s two-parameter model [42] using only positions with >95 % coverage. Bootstrap tests were carried out with 500 replicates. The additional 16S rDNA sequences were: *Bacillus subtilis* 168T (X60646), *Bacillus licheniformis* DSM 13T (X68416), *Bacillus megaterium* IAM 13418T (D16273), *Bacillus sp.* BD-87 (AF169520), *Bacillus sp.* BD-99 (AF169525), *Bacillus sp.* NRS-1691 (AF169531), *Bacillus sp.* NRS-1693 (AF169533), *Solibacillus silvestris* StLB046 (NR_074954), *Bacillus sp.* NRS-250 (AF169536), *Bacillus sp.* B-1876 (AF169494), *Bacillus sp.* NRS-1198 (AF169528), *Bacillus sp.* B-4297 (AF169507), *Bacillus sp.* NRS-111 (AF169526), *Bacillus sp.* B-183 (AF169493), *Lysinibacillus sphaericus* B-23268T (AF169495), *Lysinibacillus sphaericus* JG-A12 (AM292655), *Bacillus sp.* B-14905 (AF169491), *Lysinibacillus sphaericus* ZC1 (NZ_ADJR01000054.1:1-1487), *Bacillus sp.* B-14865 (AF169490), *Lysinibacillus fusiformis* ATCC-7055 (AJ310083), *Bacillus sp.* B-14957 (AF169492) and *Bacillus sp.* B-23269 (AF169496).

### Genome comparison

MAUVE software [23] was used in order to perform whole genome alignments and synteny comparisons. Genomes were also compared with BRIG [24] and the genomic islands and toxin-encoding genes, previously predicted, were mapped in this comparison. Dot plots were generated by Gepard [22] using the ordered contigs produced by MAUVE for each genome.

### Identification of genomic islands

Genomic islands were predicted in the complete genomes of toxic *L. sphaericus* strains using Island Viewer 3 [43]. This tool integrates the IslandPick, IslandPath.DIMOB and SIGI-HMM algorithms.

### Average nucleotide identity, correlation indexes, and DDH estimates

The values for ANIb, ANIm and Tetra were calculated by JSpecies [32] for all the possible strain pairs among *L. sphaericus* genomes. DDH estimates were obtained from Genome to Genome Distance Calculator 2.1, which transforms the distances from the high-scoring segment pairs to values analogous to DDH using a generalized linear model. This model is inferred from an empirical reference dataset comprising real DDH values and genome sequences [26]. All of the results above were represented as heatmaps using R statistical software [44].

### Pan- and core-genome analysis

The pan- and core-genomes for all strains of *L. sphaericus* as well as for the toxic and non-toxic strains were obtained using OrthoMCL [45] and Roary (with codon aware alignment) [27]. Roary uses FastTree 2.0 algorithm to infer an approximately-maximum-likelihood tree from large alignments by the Jukes-Cantor model for nucleotide evolution [27]. The results from Roary were visualized by Phandango [46] as a phylogenetic tree of core genes and by R statistical package as graphs of number of genes vs number of genomes. Orthologous gene clusters were identified and visualized by OrthoVenn which follows a similar pipeline to OrthoMCL [47].

## Additional files

Additional file 1: Figure S1. Shared and unique COGs. The Venn diagrams indicate the number of shared and unique COGs across representative toxic, non-toxic and a mixed group of strains. (PNG 251 kb) Additional file 2: Figure S2. New and unique genes in *L. sphaericus* pan-genome. The curves depict new and unique genes found with the addition of new genome sequences for toxic and for the complete set of analyzed strains. (PNG 140 kb) Additional file 3: Table S1. TETRA, ANIb and DDH estimates. The matrices indicate the value for indices TETRA, ANIb, and ANIm, as well as DDH estimates for all the studied *L. sphaericus* strains. Intra species values are colored as green and interspecies values, as red. Orange color indicates the species boundary where further analyses are required. Numbers in brackets represent the nucleotides used as seed for the respective BLAST search. Threshold values were used as indicated by Rosselló-Móra and Amann [25]. (XLSX 17 kb)
