# Supplementary figures and images for: Comparative genomics reveals Lysinibacillus sphaericus group comprises a novel species
Source: BMC Genomics. 2016 Sep 5;17(1):709. doi: 10.1186/s12864-016-3056-9 (PMC5011910; doi:10.1186/s12864-016-3056-9)

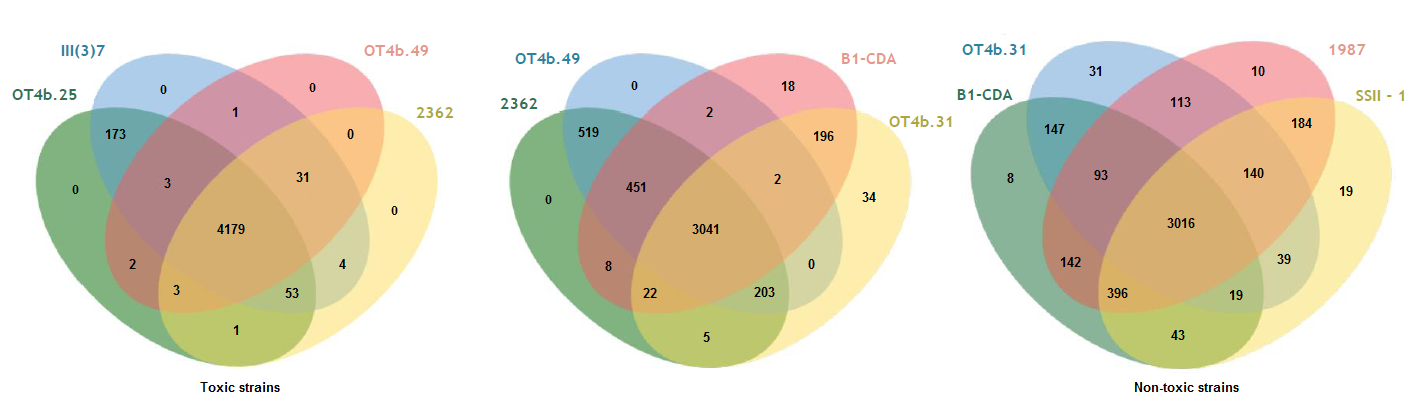

Supplement: Additional file 1: Figure S1. — Shared and unique COGs. The Venn diagrams indicate the number of shared and unique COGs across representative toxic, non-toxic and a mixed group of strains. (PNG 251 kb) [file 12864_2016_3056_MOESM1_ESM.png]

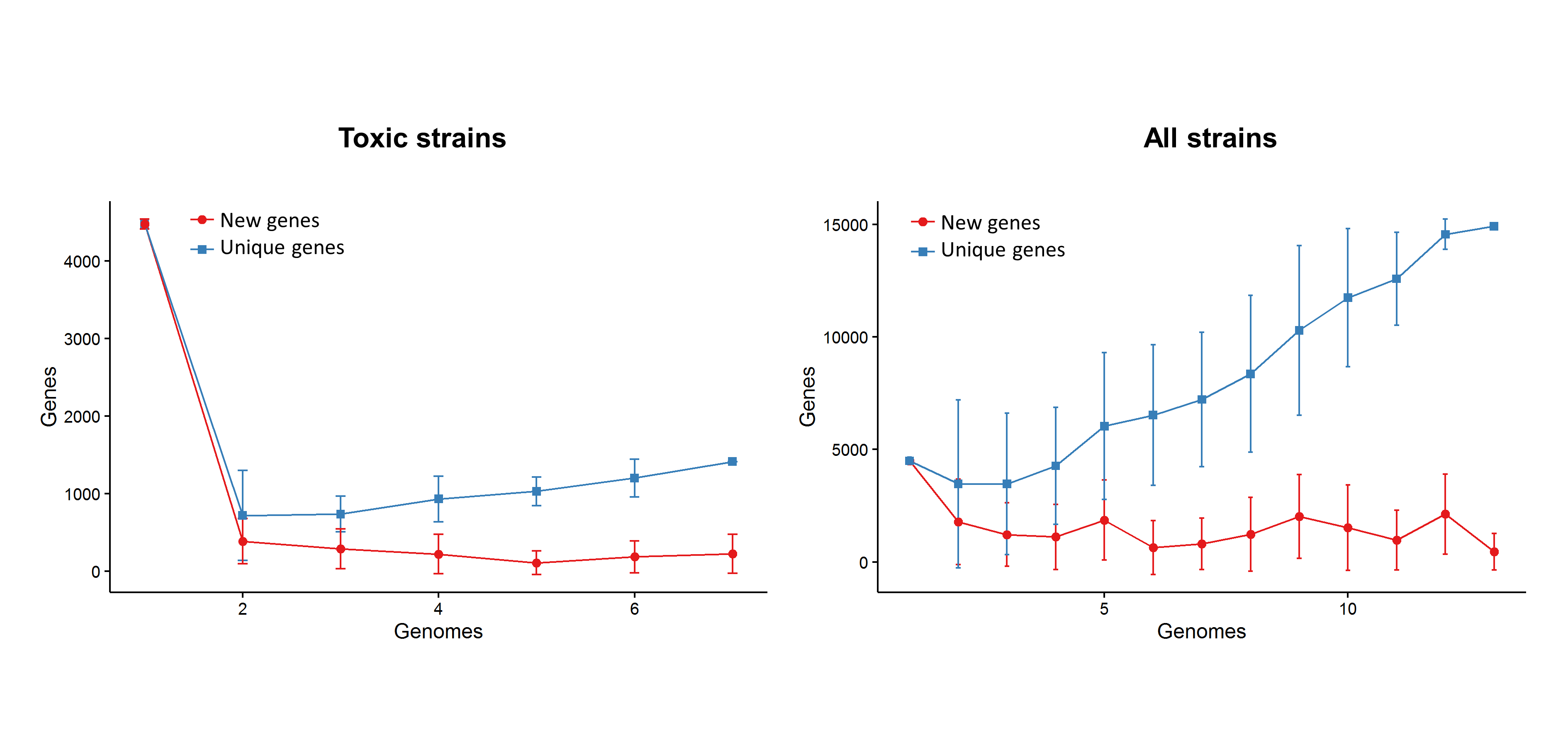

Supplement: Additional file 2: Figure S2. — New and unique genes in L. sphaericus pan-genome. The curves depict new and unique genes found with the addition of new genome sequences for toxic and for the complete set of analyzed strains. (PNG 140 kb) [file 12864_2016_3056_MOESM2_ESM.png]
